# The selective HDAC6 inhibitor Nexturastat A induces apoptosis, overcomes drug resistance and inhibits tumor growth in multiple myeloma

**DOI:** 10.1042/BSR20181916

**Published:** 2019-03-22

**Authors:** Xiaoyang Sun, Yu Xie, Xiaoshen Sun, Yao Yao, Hujun Li, Zhenyu Li, Ruosi Yao, Kailin Xu

**Affiliations:** 1 Blood Diseases Institute, Xuzhou Medical University, Xuzhou, Jiangsu, China; 2 Department of Hematology, The Affiliated Hospital of Xuzhou Medical University, Xuzhou, Jiangsu, China

**Keywords:** apoptosis, drug resistance, multiple myeloma, Nexturastat A, p21

## Abstract

Multiple myeloma (MM) is a hematological malignancy of plasma cells that produce a monoclonal immunoglobulin protein. Despite significant advances in the treatment of MM, challenges such as resistance to therapy remain. Currently, inhibition of histone deacetylases (HDACs) is emerging as a potential method for treating cancers. Numerous HDAC inhibitors are being studied for the use in monotherapy or in conjunction with other agents for MM. In the present study, we investigated the anti-myeloma effect of Nexturastat A (NexA), a novel selective HDAC6 inhibitor. We found that NexA impaired MM cells viability in a dose- and time-dependent manner. NexA also provoked a cell cycle arrest at the G1 phase in MM cells*.* Furthermore, NexA promoted apoptosis of MM cells via transcriptional activation of the p21 promoter, which may through its ability to up-regulate the H3Ac and H4Ac levels. Additionally, NexA could overcome bortezomib (BTZ) resistance in MM cells, and NexA in combination with BTZ had stronger efficacy. We also confirmed that NexA inhibited tumor growth in murine xenograft models of MM. These interesting findings provided the rationale for the future advancement of this novel HDAC6 inhibitor as a potential therapeutic anti-myeloma agent.

## Introduction

Multiple myeloma (MM) is a hematological malignancy characterized by proliferation of clonal terminally differentiated B cells (plasma cells) that secrete monoclonal immunoglobulins [1]. It is the second most common hematological malignancy after non-Hodgkin lymphoma [2]. With novel effective therapeutic strategies and improved supportive care introduced, the overall survival of patients with MM has been prolonged in recent years [1,3]. Nonetheless, most patients suffer relapses or become refractory to therapy eventually. Novel agents with higher efficacy and lower toxicity are needed to overcome drug resistance and improve therapy in MM.

Epigenetic changes, including DNA and histone modification, are recognized as participating in the development and progression of cancers [4]. Among them, acetylation and deacetylation carried out by histone acetyltransferases (HATs) and histone deacetylases (HDACs) are characterized modifications of histones. Given the key biological function of HDACs, there is an increasing interest in the potential of HDACs as relevant therapeutic targets in cancer, including MM. Various HDAC inhibitors are being investigated as treatment agents for patients with MM [5]. For example, panobinostat, vorinostat, and romidepsin have all been demonstrated to possess preclinical and clinical activity when used not only as monotherapy but also in combination with other agents including proteasome inhibitors and immunomodulatory drugs in MM [6]. In fact, panobinostat, a non-selective HDAC inhibitor, was approved for the treatment of relapsed/refractory MM by the US Food and Drug Administration (FDA) in February 2015 [7]. Although the use of HDAC inhibitors has sparked the enthusiasm of researchers, a large amount of theoretical and practical work still needs to be done in order to further explore their mechanisms of action.

Nexturastat A (NexA) is a selective inhibitor of HDAC6, which has been proved to mediate anti-melanoma effect [8,9]. Since its role on MM was rarely covered, we sought to determine if it might also possess anti-myeloma effect. In the present work, we found that NexA inhibited the viability of RPMI-8226 and U266 cell lines in a dose- and time-dependent manner. NexA caused cell cycle arrest at the G1 phase in both cell lines. Additionally, NexA promoted cell apoptosis by transcriptional activation of the p21 promoter, which may through its ability to up-regulate the H3Ac and H4Ac levels. Moreover, NexA contributed to overcome BTZ resistance. We also demonstrated that NexA inhibited tumor growth in murine xenograft models of MM. The results of our study suggested the potential clinical application of NexA as a promising therapeutic approach to treat MM.

## Materials and methods

### Reagents and cell culture

NexA was purchased from Selleck (Houston, TX, U.S.A.). The NexA stock solution was prepared by dissolving the compound in dimethyl sulfoxide (DMSO; Sigma Aldrich, St. Louis, MO, U.S.A.). The human multiple myeloma cells RPMI-8226 and U266 were obtained from American Type Culture Collection (Manassas, VA, U.S.A.). RPMI8226/BTZ100 cells were kindly provided by Dr Jacqueline Cloos (VU University Medical Center, The Netherlands). All the cell lines were cultured in RPMI-1640 medium, supplemented with 10% heat-inactivated fetal bovine serum in a humidified atmosphere of 95% air and 5% CO_2_ at 37°C.

### Cell viability assay

Cells were seeded at 1 × 10^4^ cells per 96-well plate and treated with different concentrations of NexA for 48 h or for different time points in the presence of 30 µM NexA. Cell viability was detected by the CCK-8 cell proliferation kit (Beyotime, Shanghai, China) according to the manufacturer’s instructions and the absorbance at 450 nm was read using a Microplate reader (Synergy H1, BioTek, Winooski, VT, U.S.A.).

### Western blot

Cells were lysed in RIPA lysis buffer with 1 mM phenylmethylsulfonyl fluoride (PMSF) to obtain total protein. Protein extracts were electrophoresed on SDS polyacrylamide gels and transferred to polyvinylidene fluoride (PVDF) membranes followed by blocking with 5% non-fat dry milk in Tris-buffered saline and 0.1% Tween (TBST) at room temperature. The membranes were incubated overnight at 4°C with primary antibodies diluted in blocking solution. After washing in TBST, the corresponding horseradish peroxidase-conjugated secondary antibodies were added for 1 h. Protein bands were visualized by chemiluminescence using Clarity Western ECL substrate (Bio-Rad, Hercules, CA, U.S.A.). Antibodies against β-actin, PARP1, p21, CDK2, Histone-H3, α-Tubulin and acetylated α-Tubulin were purchased from Proteintech. Antibodies against Caspase-3 and Caspase-9 were from Cell Signaling Technology. Antibodies against Histone H3Ac and Histone H4Ac were from Active Motif.

### Flow cytometric assay

To assess the distribution of nuclear DNA content, cells were treated with indicated concentrations of NexA for 48 h and fixed overnight in 75% ethanol at 4°C. Then cells were treated with 20 units/ml RNAase for 15 min at 37°C and stained with 50 µg/ml propidiumiodide (PI) prior to analysis using flow cytometry. For the determination of apoptotic cells, cells were treated with different doses of NexA for 48 h. The apoptotic cells were measured with an Annexin V-fluorescein isothiocyanate (FITC)/PI apoptosis detection kit (KeyGEN, Nanjing, China). The rate of apoptosis was detected by flow cytometry.

### RNA extraction, reverse transcription and real-time PCR

Total RNA from MM cells was isolated using the Trizol reagent (Takara, Shiga, Japan) following manufacturer’s instructions. The obtained RNA was reverse-transcribed to synthesize the complementary DNA using random primers and the Reverse Transcription System (Promega, Madison, WI, U.S.A.). Real-time PCR was carried out on a Roche Light Cycler480 using SYBR Green Real-time PCR Master Mix (Toyobo, Osaka, Japan). The relative quantitation of each gene was calculated using 2^−ΔΔCT^.

### Luciferase reporter assay

The luciferase reporter gene plasmid pGL4.20-p21 was transfected into target cells. After 8 h, cells were treated with NexA for 48 h. Then cells were collected and analyzed using the Dual Luciferase Reporter Assay System kit (Promega) according to the manufacturer’s protocol. Luciferase activity of cell lysates was determined luminometrically using the GLOMAX 20/20 luminometer (Promega) as specified by the manufacturer.

### Murine model

To evaluate the efficiency of NexA *in vivo*, SCID beige mice (3–4 weeks old, Beijing Vital River Laboratory Animal Technology Company Limited, Beijing, China) were inoculated subcutaneously with 1×10^7^ RPMI-8226 cells into the right armpit. When tumors were measurable (Day 0), mice were randomly assigned to two groups, each consisting of 5 mice. The treatment group received NexA every two days. The stock solution of NexA in DMSO was diluted in phosphate-buffered saline (PBS). The control group received an equal volume of DMSO diluted in PBS. The longest perpendicular tumor diameters were measured by a caliper every four days. Mice were sacrificed at 20 days after NexA treatment. Tumor volume was calculated by the formula: 4/3π×(width/2)^2^×(length/2). All the animal studies performed here were reviewed and approved by the Animal Ethics Committee at Xuzhou Medical University, Xuzhou, China.

### Statistical analysis

All experiments were performed independently at least three times. Data were presented as means ± standard deviation as indicated. Student’s *t*-two tail test was applied to determine statistical differences between groups. Statistically significant differences were defined as *P* < 0.05. All statistical analyses were performed using the GraphPad Prism5 software.

## Results

### NexA suppressed viability and induced G1 phase arrest of human MM cells

**Figure 1:**
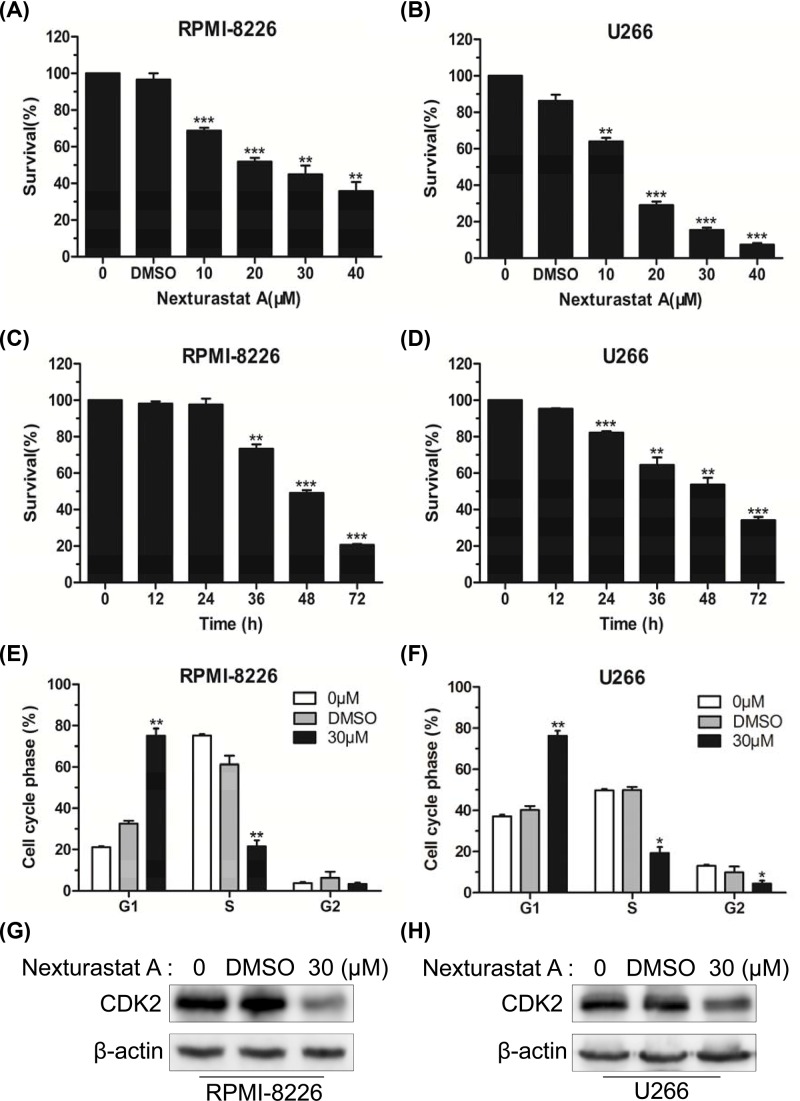
NexA suppressed viability and induced G1 phase arrest of human MM cells (**A** and **B**) The CCK-8 assay was used to detect the cell viability of RPMI-8226 and U266 cells treated with gradually increasing concentrations of NexA for 48 h. (**C** and **D**) RPMI-8226 and U266 cells were treated with 30 µM NexA for varying lengths of time, and the cell viability was analyzed by CCK-8 assay. (**E** and **F**) Percentages of the subpopulation of RPMI-8226 and U266 cells in different cell cycle phases were measured following exposure to 30 µM NexA for 48 h. Error bars indicate mean ± SD; *P < 0.05, **P < 0.01, ***P < 0.001. (**G** and **H**) Western blot showed the protein levels of CDK2 after treatment with 30 µM NexA for 48 h.

To evaluate the effect of NexA on the cell viability *in vitro*, RPMI-8226 and U266 cells were treated with gradually increasing concentrations of NexA for 48 h. The CCK8 viability assay revealed that NexA dose-dependently impaired the viability of the two cell lines (Figure 1A,B). NexA also showed a time-dependent response in inhibiting RPMI-8226 and U266 cells viability (Figure 1C,D), but it did not exert a significant anti-survival activity until 36 h after treatment in RPMI-8226 cells.

To understand the growth inhibition effect of NexA on MM cells, flow cytometry was performed to analyze cell cycle distribution in RPMI-8226 and U266 cells. The collected data demonstrated that the percentage of cells arrested in G1 phase increased in the group treated with 30 µM NexA, while that in the S phase declined. The percentage of cells in G2 phase remained stable in RPMI-8226 cells but decreased slightly in U266 cells (Figure 1E,F). We performed Western blot to examine the change in the level of Cyclin-dependent kinase 2 (CDK2). It was noticed that NexA diminished the expression of CDK2 in both cell lines (Figure 1G,H).

### NexA induced cell apoptosis in human MM cells

**Figure 2:**
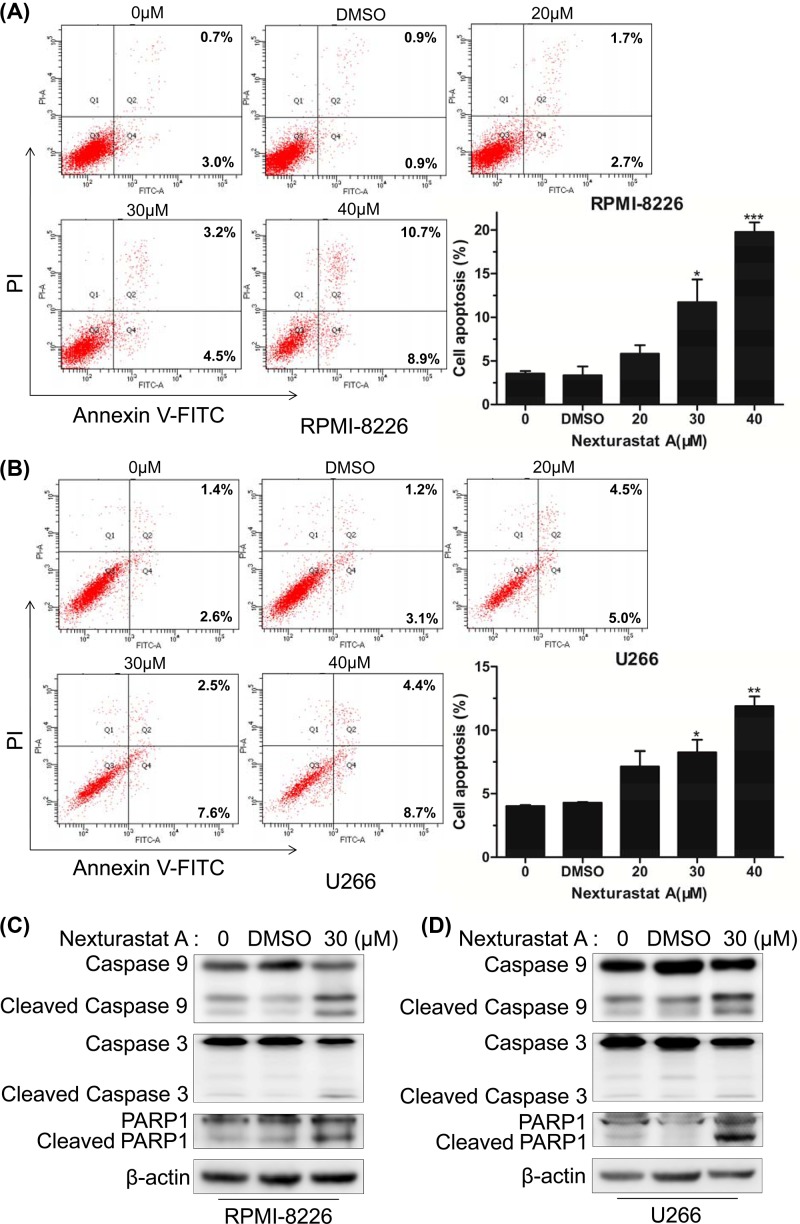
NexA induced cell apoptosis in human MM cells (**A** and **B**) Apoptosis in RPMI-8226 and U266 cells was analyzed by Annexin V-FITC/PI double-staining flow cytometry after treatment with various concentrations of NexA for 48 h. Histograms are representative of three independent experiments. Error bars indicate mean ± SD; *P < 0.05, **P < 0.01, ***P < 0.001. (**C** and **D**) Apoptosis-associated protein expression levels in RPMI-8226 and U266 cells treated with 30 µM NexA for 48 h were shown by Western blot.

To investigate the apoptosis-inducing effect of NexA on human MM cells, we examined cell apoptosis in RPMI-8226 and U266 cells using dual staining with PI and Annexin V-FITC. The two cell lines were treated with different concentrations of NexA for 48 h. Flow cytometry analysis showed increases of the percentage of apoptotic cells in a dose-dependent manner in both cell lines (Figure 2A,B). The detection of apoptosis-associated proteins demonstrated that NexA treatment led to the cleavage of Caspase3, Caspase9 and PARP1 in both cell lines (Figure 2C,D). These data indicated that NexA effectively elicits apoptosis of MM cells.

### NexA contributed to overcome bortezomib resistance for human MM cells

**Figure 3:**
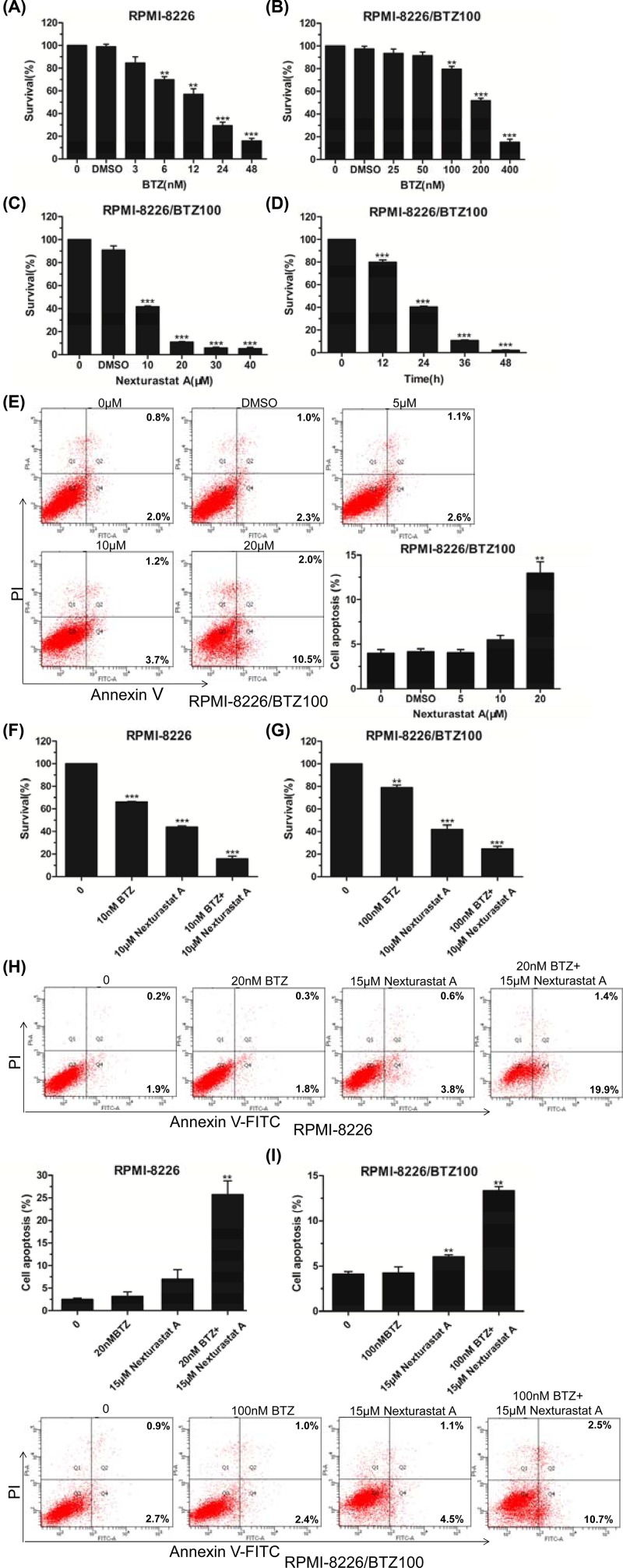
NexA contributed to overcome bortezomib resistance for human MM cells (**A** and **B**) BTZ-resistance in RPMI-8226/BTZ100 cells relative to RPMI-8226 cells after 48-h BTZ exposure was confirmed by CCK8 assay. (**C**) RPMI-8226/BTZ100 cells grew with different doses of NexA for 48 h and then the cell viability was analyzed. (**D**) RPMI-8226/BTZ100 cells were cultured in the presence of 30 µM NexA for different periods of time and then the cell viability was detected. (**E**) Flow cytometry analysis of the ratio of apoptosis in RPMI-8226/BTZ100 cells treated with various concentrations of NexA (0, DMSO, 5, 10, 20 µM) for 48 h. Histograms are representative of three independent experiments. (**F** and **G**) RPMI-8226 and RPMI-8226/BTZ100 cells grew with NexA and/or bortezomib for 48 h and then the cell viability was analyzed. (**H** and **I**) Flow cytometric detection of the percentage of apoptotic cells in RPMI-8226 and RPMI-8226/BTZ100 cells treated with NexA and/or bortezomib. Histograms are representative of three independent experiments. Error bars indicate mean ± SD. **P < 0.01, ***P < 0.001.

Bortezomib (BTZ) has been successfully applied in the treatment of MM over the last decade. While the clinical benefit of BTZ in MM remains unchallenged, the extensive occurrence of resistance imposes restrictions on the long-term utility [10]. RPMI-8226/BTZ100 cell lines grow in the presence of 100 nM BTZ. The 96-h IC50 value of RPMI-8226/BTZ100 cells toward BTZ was demonstrated to be 105.9 ± 14.9 nM by cytotoxicity assay [11]. We confirmed BTZ-resistance in RPMI-8226/BTZ100 cells relative to RPMI-8226 cells after 48-h BTZ exposure. Cell viability assay showed the 48-h IC50 values toward BTZ to be 12.89 nM in RPMI-8226 cells and 194.9 nM in RPMI-8226/BTZ100 cells (Figure 3A,B). Subsequently, we conducted CCK8 assays to detect the inhibitory effects of NexA on RPMI-8226/BTZ100 cell lines. The data indicated that the viability of RPMI-8226/BTZ100 cells was remarkably suppressed by NexA in a dose- and time-dependent manner (Figure 3C,D). Furthermore, induction of apoptosis was detectable in RPMI-8226/BTZ100 cells after 48-h exposure to NexA even at concentration of 20 µM (Figure 3E). We also examined whether BTZ in combination with NexA could improve the efficacy of BTZ in MM cells. We found that 10 and 100 nM BTZ alone inhibited cell growth of RPMI-8226 cells and RPMI-8226/BTZ100 cells, respectively, and the inhibition was further enhanced if they were used in combination with 10 µM NexA (Figure 3F,G). Moreover, 20 and 100 nM BTZ treatment alone had no distinct apoptosis-inducing effects in RPMI-8226 cells and RPMI-8226/BTZ100 cells, respectively, whereas there were notable increases in percentage of apoptotic cells with 15µM NexA (Figure 3H,I). Taken together, these data strongly suggested that NexA contributes to overcome BTZ resistance for human MM cells.

### NexA promoted apoptosis of human MM cells via transcriptional activation of the p21 promoter

**Figure 4:**
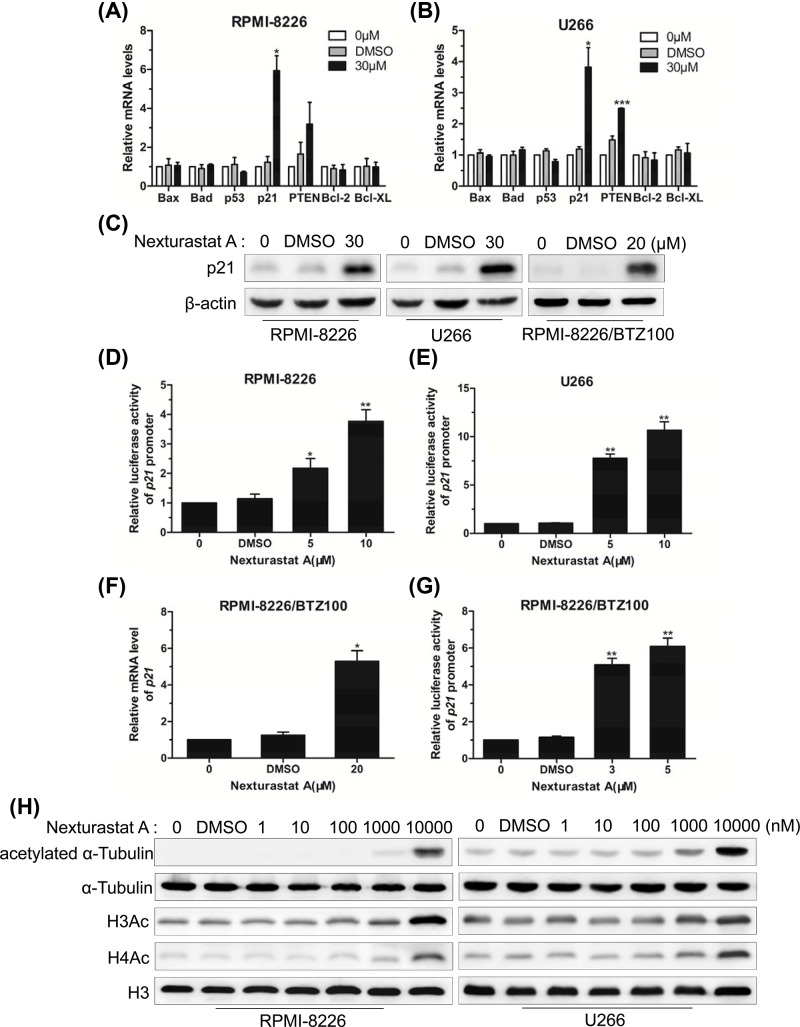
NexA promoted apoptosis of human MM cells via transcriptional activation of the p21 promoter (**A** and **B**) The expression levels of apoptosis-related factors of RPMI-8226 and U266 cells cultured in the presence of 30 µM NexA for 48 h were estimated by real-time PCR. (**C**) The protein expression level of p21 was evaluated by Western blot. β-Actin was used as internal control. (**D** and **E**) Luciferase reporter gene assay of p21 promoter activity in RPMI-8226 and U266 cells treated with different doses of NexA for 48 h. (**F**) The mRNA level of p21 in RPMI-8226/BTZ100 cells treated with 20 µM NexA for 48 h was estimated by real-time PCR. (**G**) Luciferase reporter gene assay of p21 promoter activity in RPMI-8226/BTZ100 cells treated with different doses of NexA for 48 h. Error bars indicate mean ± SD; *P < 0.05, **P < 0.01, ***P < 0.001. (**H**) The acetylation status of histone H3, histone H4 and α-tubulin in MM cells treated with different doses of NexA for 48 h was detected by Western blot.

To figure out the internal molecular mechanism of NexA-inducing apoptosis of MM cells, the expression levels of apoptosis-related factors were estimated utilizing real-time PCR. The results showed that the p21 mRNA levels were higher in RPMI-8226 and U266 cells treated for 48 h (Figure 4A,B). Then Western blot assay manifested that NexA treatment also resulted in evident increases in p21 protein levels in both cell lines (Figure 4C). After which we carried out p21 luciferase reporter gene assays to determine whether NexA could enhance the promoter activity of p21 accordingly. The data indicated the enhanced activity of p21 with 5 and 10 µM NexA for both cell lines (Figure 4D,E). We also evaluated p21 induction in RPMI8226/BTZ100 cells. As shown in Figure 4C,F, p21 mRNA and protein levels increased after cells were treated with 20 µM NexA. We observed enhanced p21 promoter activity in RPMI8226/BTZ100 cells treated with 3 and 5 µM NexA (Figure 4G).

Now that NexA is a HDAC6 inhibitor, its ability to regulate gene expression may depend on histone acetylation. Besides histone deacetylase activity, HDAC6 possesses α-tubulin deacetylase activity [12]. Western blot analysis was performed to detect the acetylation status of histone H3 and H4, as well as α-tubulin in MM cells. Interestingly, the increases in α-tubulin acetylation were not found until concentrations of 1 and 10 μM were used. The acetylation of histone H3 and H4 also increased with 1 and 10 μM NexA in MM cells (Figure 4H).

### NexA inhibited tumor growth in murine xenograft models

**Figure 5:**
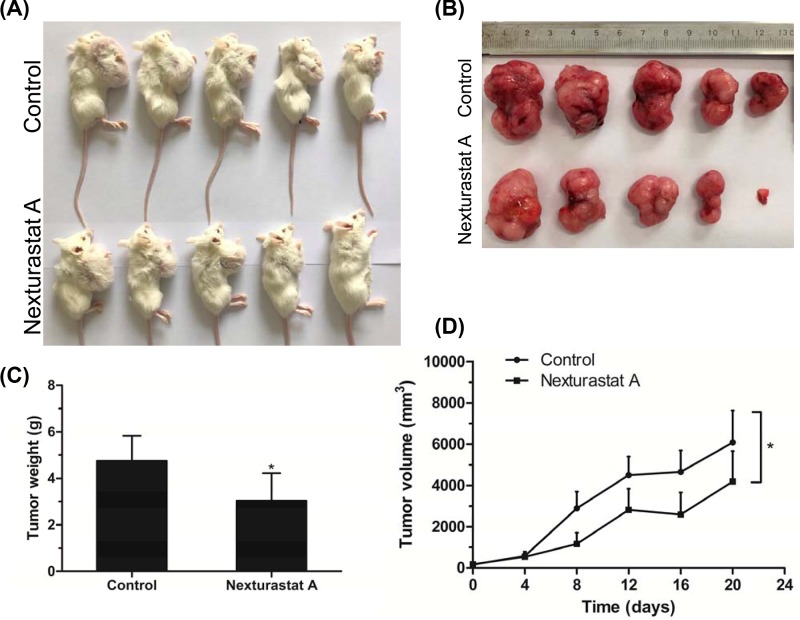
NexA inhibited tumor growth in murine xenograft models SCID beige mice were inoculated subcutaneously with 1 × 10^7^ RPMI-8226 cells into the right armpit. Tumors formed at the site of injection in all mice. (**A**) Photographs of mice killed at day 20. (**B**) Photographs of xenograft tumors harvested at day 20. (**C**) Tumor weights were obtained at the study end point. (**D**) The tumor growth curves of the control group and the NexA group during the whole experiment. Error bars indicate mean ± SD. *P < 0.05.

The anti-tumor activity of NexA on MM cells *in vivo* was further examined in murine xenograft models of MM. There was tumor formation at the site of injection in all mice after subcutaneous inoculation of RPMI-8226 cells. After 20 days of treatment, mice were killed and tumors were collected (Figure 5A,B). As shown in Figure 5C, NexA treatment resulted in reductions of tumor weight as compared with the control group. In the meanwhile, shrinkage of tumor size was detected in mice treated with NexA (Figure 5D). These results indicated that NexA reduces the growth of MM xenografts in SCID beige mice.

## Discussion

Epigenetic changes are increasingly viewed as key events in the initiation and progression of tumor. The effects of HDACs on a variety of cellular processes make HDACs the promising target for the development of novel anti-myeloma agents. Especially, emerging studies have focused on inhibitors selective for HDAC isotypes as they could circumvent unwanted toxicity related to the off-target effects of pan-HDAC inhibitors. It has been reported that HDAC6-selective inhibition can bring on increased sensitivity of transformed cells to certain anti-cancer agents [13]. Furthermore, a series of studies of selective HDAC6 inhibitors showed significant anti-MM activity alone and in combination with other agents [14–16].

In the present study, we investigated the anti-myeloma effect of NexA, a novel selective HDAC6 inhibitor. Our results indicated that NexA could result in decreased viability of MM cells in a dose- and time-dependent manner. We assumed that the reduction of cell growth is attributable to inhibition of the cell cycle and/or induction of apoptosis. Hence, the cell cycle distribution and apoptosis in MM cells were analyzed. We discovered that NexA had an obvious impact on the cell cycle, as it provoked a cell cycle arrest in G1 phase. Previous published data have suggested that CDK2 was required during the G1 to S phase transition [17,18]. A decline of CDK2 at the protein level was detectable after NexA treatment. We also confirmed the apoptosis-inducing effect of NexA. It triggered apoptosis of MM cells in a concentration-dependent manner. Meanwhile, Caspase 9, Caspase 3, and PARP1 were proteolytically activated. Caspase-9 and -3 belong to caspase family of cysteine proteases that has been regarded as critical participants in the process of apoptosis. Once activated, caspase-9 went on to cleave caspase-3, initiating the caspase cascade [19]. Caspase-3 also cleaved the death substrate PARP during apoptosis.

Our discovery that p21 transcription was strongly induced brought insight into the possible action mechanism of NexA. p21, one of the major tumor suppressors, is a crucial factor in the regulation of cell proliferation, cycle, and apoptosis. Known as CDK-interacting protein1, p21 is a member of families of CDK inhibitors [20]. As a universal inhibitor of the cyclin/CDK complexes [20], the higher expression of p21 was in accord with the lower level of CDK2 after NexA treatment. With respect to cell proliferation and apoptosis, the molecular behavior of p21 appears to be cell and environment dependent. It has been shown that p21 may induce or prevent apoptosis depending on cell type, inducing stimuli, subcellular localization, and so forth [21,22]. More importantly, it has been reported that a variety of anti-tumor drugs, including HDAC inhibitors, exerted anti-cancer effects by inducing p21 expression [23]. In this research, we also found that p21 promoter was transcriptional activated in NexA-induced apoptosis of MM cells. We observed enhanced p21 promoter activity in RPMI-8226 and U266 cells treated with 5 and 10 µM NexA, as well as in RPMI8226/BTZ100 cells treated with 3 and 5 µM NexA. However, higher doses of NexA treatment gave inconsistent results with this assay, presumably due to excessive growth inhibition and apoptosis of cells after treatment. As a HDAC6 inhibitor, NexA may regulate gene expression depending on histone acetylation. NexA was demonstrated selective inhibitory activity against HDAC6 with an enzymatic IC50 value of 5 nM [8]. NexA was 600-, 1380-, and 1330-fold less active against HDAC1, 2 and 3 (Class I HDAC), respectively [8]. Surprisingly, NexA did not show obvious selective inhibitory activity against HDAC6 in detection of the acetylation status of HDAC substrates in MM cells.

Since being approved for clinical use in 2003, BTZ has afforded great benefits to patients with MM, and BTZ-based therapies have become a major method for the MM treatment. However, BTZ resistance emerged as an important clinical problem. The present study suggested that many HDAC inhibitors were able to synergize with proteasome inhibitors and overcome BTZ-induced resistance in MM [24–26]. Our study manifested that RPMI-8226/BTZ100 cells were even more sensitive to NexA than RPMI-8226 and U266 cells. Moreover, NexA in combination with BTZ had stronger efficacy in MM cells.

In conclusion, NexA reduced MM growth *in vitro* and *in vivo*, arrested MM cells at G1 phase, overcame BTZ resistance and induced apoptosis of MM cells by the transcriptional activation of p21. These interesting findings provide the rationale for the future advancement of this novel HDAC6 inhibitor as a potential therapeutic anti-myeloma agent.

## Abbreviations

BTZ: bortezomib
HAT: histone acetyltransferase
HDAC: histone deacetylase
MM: multiple myeloma
NexA: Nexturastat A
